# Complete Genome Sequence of CHYJ130330, a Highly Virulent Strain of Porcine Epidemic Diarrhea Virus in South China

**DOI:** 10.1128/genomeA.00165-14

**Published:** 2014-04-17

**Authors:** Aiqing Jia, Xiaosheng Feng, Qi Liu, Ruyue Zhou, Guiping Wang

**Affiliations:** Guangdong Haid Institute of Animal Husbandry & Veterinary, PanYu, Guangzhou, Guangdong, China

## Abstract

Porcine epidemic diarrhea virus (PEDV) strain CHYJ130330 was isolated from southern China and shown to be highly virulent when inoculated into neonatal pigs. This report describes the complete genome sequence of CHYJ130330. These data will provide important insights into the variation of PEDV in China.

## GENOME ANNOUNCEMENT

Porcine epidemic diarrhea virus (PEDV) can cause acute diarrhea and dehydration in 2- to 7-day-old piglets. Infected piglets die within 2 to 3 days. The mortality rate associated with this virus is 90% (1). PEDV emerged in the 1980s and has since become more serious, having resulted in severe economic losses in China (2–4).

In March 2013, the wild-type PEDV strain CHYJ130330 was obtained from a fecal specimen of a 3-day-old dead piglet on a commercial swine farm in Guangdong Province, southern China. This strain was successfully passaged in a Vero cell line. Twenty-four hours after inoculation, the Vero cells demonstrated an obvious cytopathic effect characterized by cell fusion and syncytium formation. The CHYJ130330 strain demonstrates high virulence; the oral ingestion of 10× the 50% tissue culture infective dose (TCID_50_) of this virus by 2- to 7-day-old piglets can induce severe diarrhea after 48 h.

To determine the complete-genome sequence of the PEDV isolate CHYJ130330, 21 pairs of primers based on this isolate were designed to generate overlapping amplicons by reverse transcription-PCR. The complete genome of CHYJ130330 is 28,038 nucleotides (nt) in length, with a poly(A) tail. The genomic organization of the isolate is similar to that previously described and includes a 5′ untranslated region (UTR) (nt 1 to 292), a replicase gene comprising open reading frame 1a (ORF1a) (nt 293 to 12601) and ORF1b (nt 12601 to 20637), a spike gene (nt 20634 to 24794), ORF3 (nt 24794 to 25468), an envelope gene (nt 25449 to 25679), a membrane gene (nt 25687 to 26367), a nucleoprotein gene (nt 26379 to 27704), and a 3′ UTR (nt 27705 to 28038). The complete genome of PEDV strain CHYJ130330 has nucleotide identities of 96.5% to 99.3% with other complete PEDV genomes available in GenBank (1, 2, 5–9). We observed a high nucleotide identity of 99.1% with the U.S. strain IA1 (10), which was identified on 29 April 2013 by the Iowa State University Veterinary Diagnostic Laboratory and corroborated on 16 May 2013 by the National Veterinary Services Laboratories (5). Porcine epidemic diarrhea was recognized in U.S. swine for the first time in early 2013; since then, PEDV has been detected in 18 U.S. states and has resulted in large economic losses (6, 7).

In conclusion, the PEDV isolate CHYJ130330 is a highly virulent strain that may be a candidate for vaccine development and diagnostic techniques. Investigations of its immunogenicity and pathogenicity are ongoing.

### Nucleotide sequence accession number.

The complete genome sequence of PEDV strain CHYJ130330 has been deposited in GenBank under the accession no. KJ020932.
